# Clinical outcome after knee ligament reconstruction with tendon allografts

**DOI:** 10.1186/s40634-021-00331-4

**Published:** 2021-02-07

**Authors:** Jon Olav Drogset, Kristina Hovde Størset, Thea Marie Nitteberg, Tone Gifstad

**Affiliations:** Norwegian University of Science and Technology, Trondheim University Hospital, Trondheim, Norway

**Keywords:** Knee ligament, Allograft, Surgical treatment, Ligament reconstructions

## Abstract

**Purpose:**

The purpose of this study is to investigate the clinical outcome for patients after knee ligament reconstructions with allografts at a university hospital.

**Methods:**

A total of 33 patients received allografts for reconstructive knee surgery between 2007 and 2017. The follow up evaluation consisted of a clinical knee examination including evaluation of range of motion (ROM), lateral and medial laxity, the Lachman test, the Pivot shift test, the sag test, the posterior drawer test and checking for patellofemoral pain. The following patient-reported outcome measures (PROMs) were used; the Lysholm Function Score, the Tegner activity score, and the Knee injury and Osteoarthritis Outcome Score (KOOS).

**Results:**

Twenty-one (64%) patients were available for the follow-up evaluation and the mean follow-up time was 4.8 years. A total of 16 out of 21 patients had multiligament injuries of which the ACL was the ligament most frequently ruptured. At the time of follow-up, 14 out of 16 patients (87%) with ACL injury had Lachman test grade 0 or grade 1 + , and 12 out of 13 (92%) had a pivot shift grade 0 or 1 + . The mean Lysholm Score was 74. All mean KOOS subscale values were ≥ 59 at the follow-up. The preoperative Tegner activity score was 3 (range, 1–6) and 4 (range, 2–6) at follow up. There were no deep postoperative infections. A total of 19 out of 21 patients (90%) reported that they would have undergone surgery again had they known the clinical outcome in advance.

**Conclusions:**

The patients improved from the preoperative score to the follow-up score in the knee-related Quality of Life (QoL) KOOS subscale. None of the patients were diagnosed with deep postoperative infections.

## Introduction

A multiligament injury of the knee is a serious injury which historically had variable prognosis, depending on the severity and management of the injury [15]. Current literature summarizes that surgically treated patients have far better outcomes compared to non-operative management [7, 10, 32], and that repair has a higher failure rate compared to reconstruction, especially regarding repair of the posterolateral corner [22].

Traditionally, autografts have been used for the purpose of reconstruction. Today, tendon allografts are increasingly used in certain patients and confer advantages such as quality graft with less limitations regarding size, shorter time in surgery, less scarring and reduced donor-site morbidity [15, 16, 39, 42]. In Scandinavia, allografts are mainly used in anterior cruciate ligament (ACL) revision and in reconstructions of multiligament injuries [11]. According to The Norwegian National Knee Ligament Registry (NKLR) regarding the selection of graft choices in the period 2004–2018 for all primary reconstruction, allograft was used in 1.5% of the cases. For all injuries with revision reconstruction, the equivalent number was 6.3% [33]. A study investigating the variation in graft choices between five European registries and a registry in the US including 101,125 ACL reconstructions concluded that allografts were used in 40% of all procedures investigated in the US compared with 0.3 to 6.3% in the European registries (11).

Potential challenges of allografts include difficulties with strength, availability, and cost. Regarding strength, some studies report a higher risk of revision compared to autografts [18, 26, 44]. Some hospitals in Norway currently harvest allografts for domestic use, and some purchase the allografts from suppliers abroad. Additional challenges to consider with the use of allografts are transportation time of the grafts and costs, which may influence the choice of grafts and where to purchase them. Other ethical, religious and legislative concerns may also influence the use of allografts. This includes variations in price from different suppliers, which in turn are influenced by the legislation of the current country.

There is a risk for infection associated with the use of allografts. One way to address this problem is to sterilize the grafts via gamma radiation. Gamma radiation is an effective method for making the grafts safe for clinical use [36]. There seems to be a difference between the quality of irradiated versus nonirradiated grafts. γ- irradiated allografts showed a higher failure rate than autograft and fresh frozen allograft [13]. Other studies suggest that infections might be a challenge regarding the use of allograft in reconstructive knee surgery [8, 27].

The use of allografts in knee ligament reconstruction has become a well-established alternative to autografts in certain patients. Little research has been performed in Europe on the results after the use of allografts. The aim of the present study was to evaluate the outcomes for patients who had knee ligament reconstruction with an allograft at our hospital.

## Materials and methods

Included in this retrospective study are patients who received allografts for knee ligament reconstruction at our hospital from 2007 to 2017. Information about recipients of allografts during this period was retrieved from a local hospital registry. The Regional Committee for Medical and Health Research Ethics (REC) 24.04.2019 and the Norwegian Data Inspectorate 29.07.2019 approved the study. A written consent was signed by all the participants prior to collecting relevant data and proceeding with examinations. Further information on which type of allograft patients received and what kind of reconstruction was performed were collected from the hospital electronic patient charts.

The clinical examination at follow-up included evaluation of range of motion (ROM), lateral and medial laxity, the Lachman test, the Pivot shift test, the sag test, the posterior drawer test and the Grinding`s test for patellofemoral pain. All examinations were carried out by two independent observers: a fifth-year medical student and an experienced orthopedic surgeon. In addition, the following patient-reported outcome measures (PROMs) were used postoperatively: the Lysholm Function Score [24], the Tegner activity score [38], and the Knee injury and Osteoarthritis Outcome Score (KOOS) [34]. The patients’ preoperative KOOS was retrieved from the NKLR and compared with scores collected at the follow-up evaluation. To consider general satisfaction, the patients were asked, knowing the outcome, if they would have done the surgery again. Also included were questions regarding subsequent traumas, knee injuries or knee surgeries, postoperative infections and other complications after the primary surgery.

### Allograft procurement and processing

The allografts used in our hospital are purchased from a tissue bank (Cliniques universitaires Saint-Luc, Bruxelles) in Belgium. The Belgium legislation [40] concerning traceability of the graft requires the name of the recipient of the graft before shipment. This means that no allografts can be ordered until the patients receiving them are being scheduled for surgery. On arrival, the grafts are immediately placed in a freezer at -70 °C for storage until the day of surgery.

At the day of surgery, the allografts are removed from their sterile boxes in the freezer and immersed into 4 L of sterile isotonic saline at a maximum temperature of 40 °C. Samples for cultures and resistance are collected, then 300 mg rifampicin is added to the saline according to the procedures for procurement from Belgium. The grafts are soaked in this solution for a minimum of 30 min. The surgeon prepares the graft before rinsing it with sterile saline and then implanting it.

All patients receive antibiotic prophylaxis; cefazolin 2 g × 2 or 2 g × 4 if the procedure is open surgery, and thrombosis prophylaxis; enoxaparin sodium (klexane) 20 mg × 1 subcutaneously for ten days.

### Statistical analyses

Data were received depersonalized from the NKLR. All statistical analyses were conducted with IBM SPSS Statistics version 25. Due to a limited number of patients only two variables were tested for statistically significant differences; the Wilcoxon Signed Rank Test was used to compare difference between preoperative and postoperative values for the Tegner activity score and knee-related Quality of Life (QoL) KOOS subscore. The KOOS for all five subscales at the two different time-points are presented as means with 95% confidence intervals (CI).

## Results

**Fig. 1:**
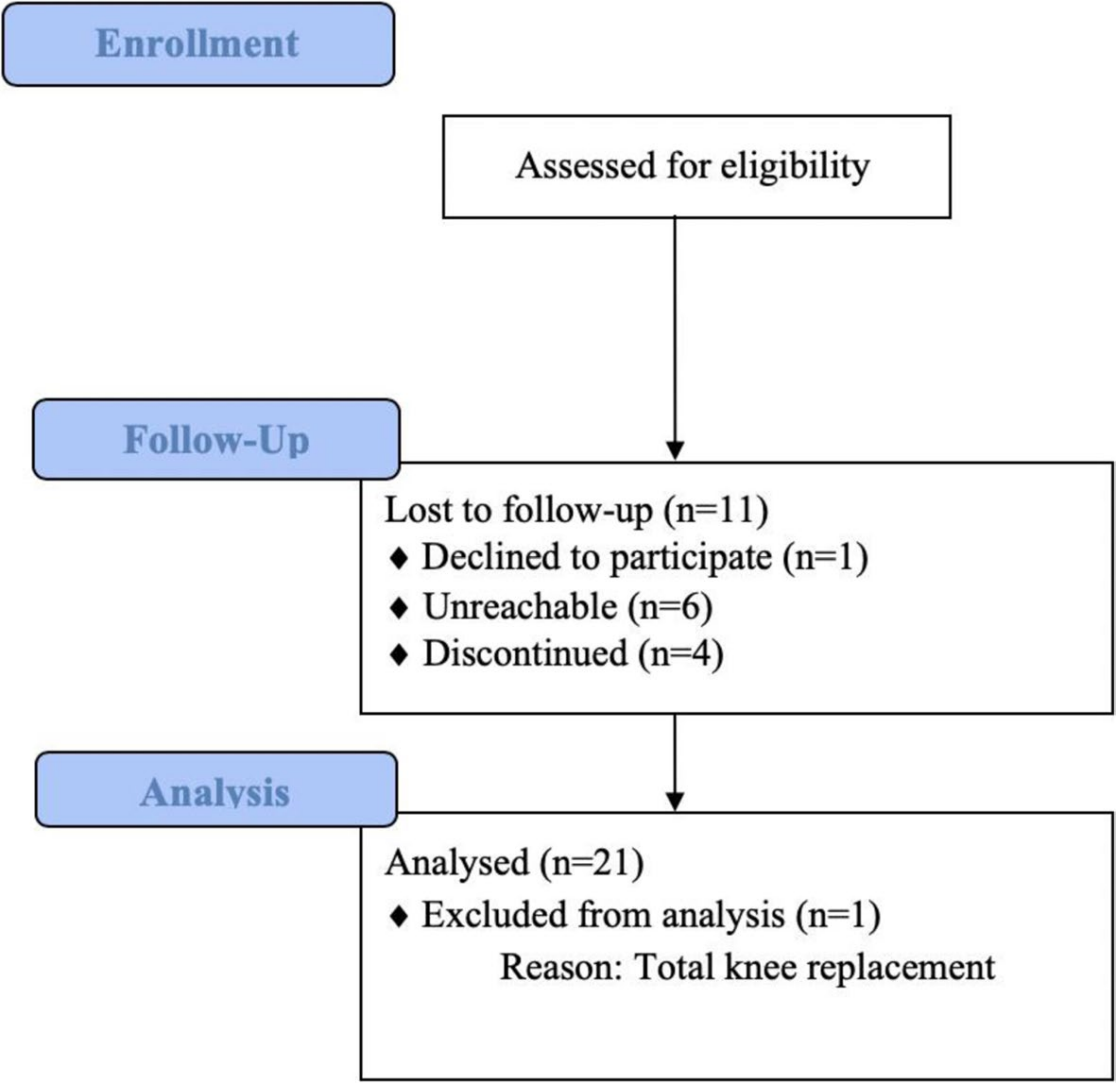
Study flowchart of the patients included in the study

Twenty-one out of thirty-three patients receiving allograft during the set period were included in the study. This is shown in a flowchart in Fig. 1. One patient had a total knee replacement, six patients were lost to follow-up, one patient refused to participate, and four patients were not able to meet for the follow-up evaluation. One patient was paraplegic, and this patient's PROMS were excluded, while the clinical examinations were included. The clinical examination results were little affected by this and were included in the results.

All twenty-one patients underwent interview and clinical examination. Twelve patients (57%) were men and thirteen had a right knee injury. Fifteen patients had open procedures. The patients median age at surgery was 41 years (range, 17–57 years) and the median time from injury to surgery was 12 months (range 5–40 months). Median age at follow-up was 46 year (range, 22–64 years) and median time from surgery to follow up was 4 years (range, 1–9 years). The injury mechanism is shown in Table 1. Thirteen patients underwent a primary ligament reconstruction with allograft. Seven patients had revision surgery, five of which were one stage procedures and two were two stage procedures. One multi trauma patient was initially stabilized with sutures and was later reconstructed. 11 patients had previous surgery in the same knee prior to the injury.

**Table 1:** The number of patients (%) in different type of activities leading to the injuries

Soccer	4 (19)
Handball	1 (4.8)
Alpine skiing	4 (19)
Cross contry skiing	3 (14)
Traffic	2 (9.5)
Fall	1 (4.8)
Other	5 (24)
Unknown	1 (4.8)

The various combinations of ligament reconstructions and grafts used are shown in Table 2. Seventeen patients had a multiligament injury with two or more ligaments ruptured. The graft types are shown in Table 3. For ACL reconstructions, Bone-Patella-Tendon-Bone (BPTB) allografts were used in 50% of the cases. In 4 of 5 patients who had a lateral collateral ligament (LCL) reconstruction, Achilles allografts were used. Graft choice varied between different allograft types regarding the medial collateral ligament (MCL) and posterior cruciate ligament (PCL) ruptures.

**Table 2:** The various combinations of ligament reconstructions and grafts used

	ACL	LCL	ACL/PCL	ACL/MCL	ACL/LCL	PCL/MCL	ACL/PCL/MCL
**TOTAL**	**3**	**1**	**2**	**2**	**4**	**4**	**5**
BPTB allograft	3		1	1	1	2	3
Achilles allograft		1	1		3		1
Hamstring allograft					1		
Quadriceps allograft				1	1		
Tibialis anticus allograft						3	3
Tibialis posticus allograft			2	1			3
Hallucis longus allograft						1	
Peroneus longus allograft + hallucis longus allograft							1
Peroneus brevis allograft							1
Triceps brachii allograft						1	
Flexor antebrachium allograft + fascia lata allograft						1	
Autograft				1	2		3

**Table 3:** The various grafts used for the different ligament injuries

	ACL	PCL	MCL	LCL
**TOTAL**	**16**	**11**	**11**	**5**
BPTB allograft	8	3		
Achilles allograft	1		1	4
Hamstring allograft	1			
Quadriceps allograft			1	1
Tibialis anticus allograft		2	4	
Tibialis posticus allograft	1	4	1	
Hallucis longus allograft			1	
Peroneus longus allograft + hallucis longus allograft			1	
Peroneus brevis allograft			1	
Triceps brachii allograft		1		
Flexor antebrachium allograft + fascia lata allograft			1	
Autograft	5	1		

The results from the evaluation of ROM, lateral and medial laxity are shown in Tables 4 and 5, respectively. The results from the Lachman test and the Pivot shift test are presented in Table 6. Seven patients with ACL injury had a Lachman grade 0 and three of them had allografts. A total of eight patients with ACL injury had a pivot shift grade 0, five of these were reconstructed with allografts. From sixteen ACL-ruptures, eleven patients had allografts (69%) and five patients (31%) had autografts. Within this group, seven patients had a grade 0 Lachman, of which four received autografts and three allografts. Out of the remaining nine patients, seven had a Lachman grade 1 where six of these had allografts and one had an autograft. The last two patients, who both had allografts, presented with grade 2 and 3 on the Lachman test. The results from posterior drawer test and sag test are shown in Table 7. Three out of twenty-one reported patellofemoral pain. One of these patients had a previously confirmed patellofemoral osteoarthrosis.

**Table 4:** The range of motion at follow-up in degrees

**Flexion deficit**	
< 10°	16
≥ 10°	5
**Extension deficit**	
< 5°	19
≥ 5°	2

**Table 5:** The lateral and medial laxity at follow-up

**MCL**	
Extended	
0	18
1	3
2	0
30°Flexion	
0	15
1	4
2	2
**LCL**	
Extended	20
0	0
1	1
2	
30°Flexion	
0	19
1	1
2	1

**Table 6:** The Lachman test and Pivot shift test at follow-up

**Lachman**	
Grade 0 and 1	19
Grade 2 and 3	2
**Pivot shift**	
Grade 0 and 1	16
Grade 2 and 3	2
Missing	3

**Table 7:** The posterior drawer test and sag test at follow-up

**Posterior drawer test**	
Grade 0 and 1	21
Grade 2 and 3	0
**Sag test**	
Grade 0 and 1	20
Grade 2 and 3	1

**Fig. 2:**
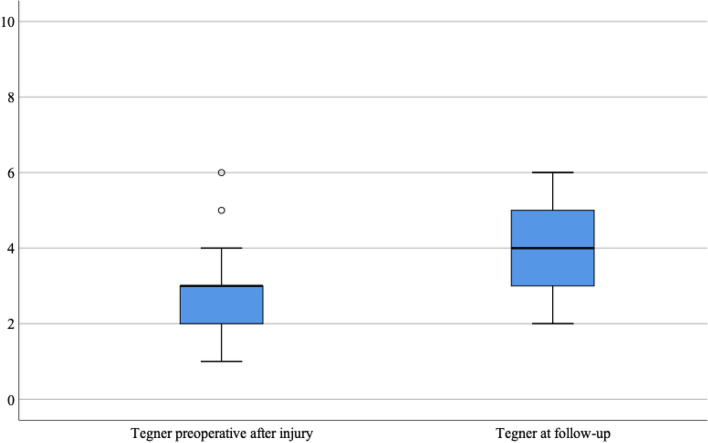
The median preoperative after injury Tegner activity score and score at the follow-up evaluation

**Fig. 3:**
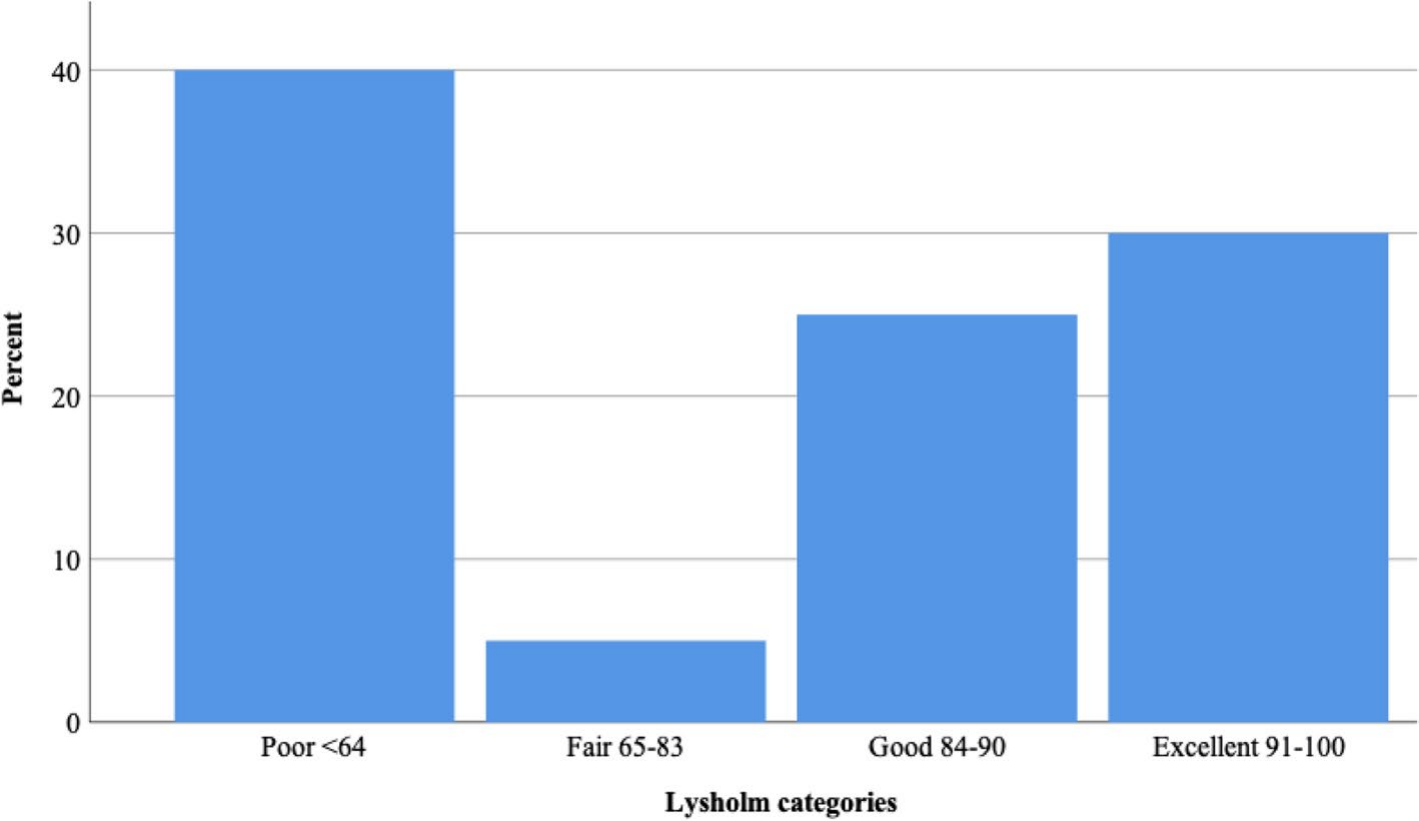
Percentage distribution of the four categories in the Lysholm function score

The median preoperative Tegner activity score was 3 (range, 1–6) and at follow up 4 (range, 2–6) (Fig. 2), *p* = 0.002. The mean Lysholm Function score was 74 (95% Cl 64–86; range, 21–100), with a median of 85 at follow-up. The distribution in the different categories (poor, fair, good, excellent) are shown in Fig. 3.

**Fig. 4:**
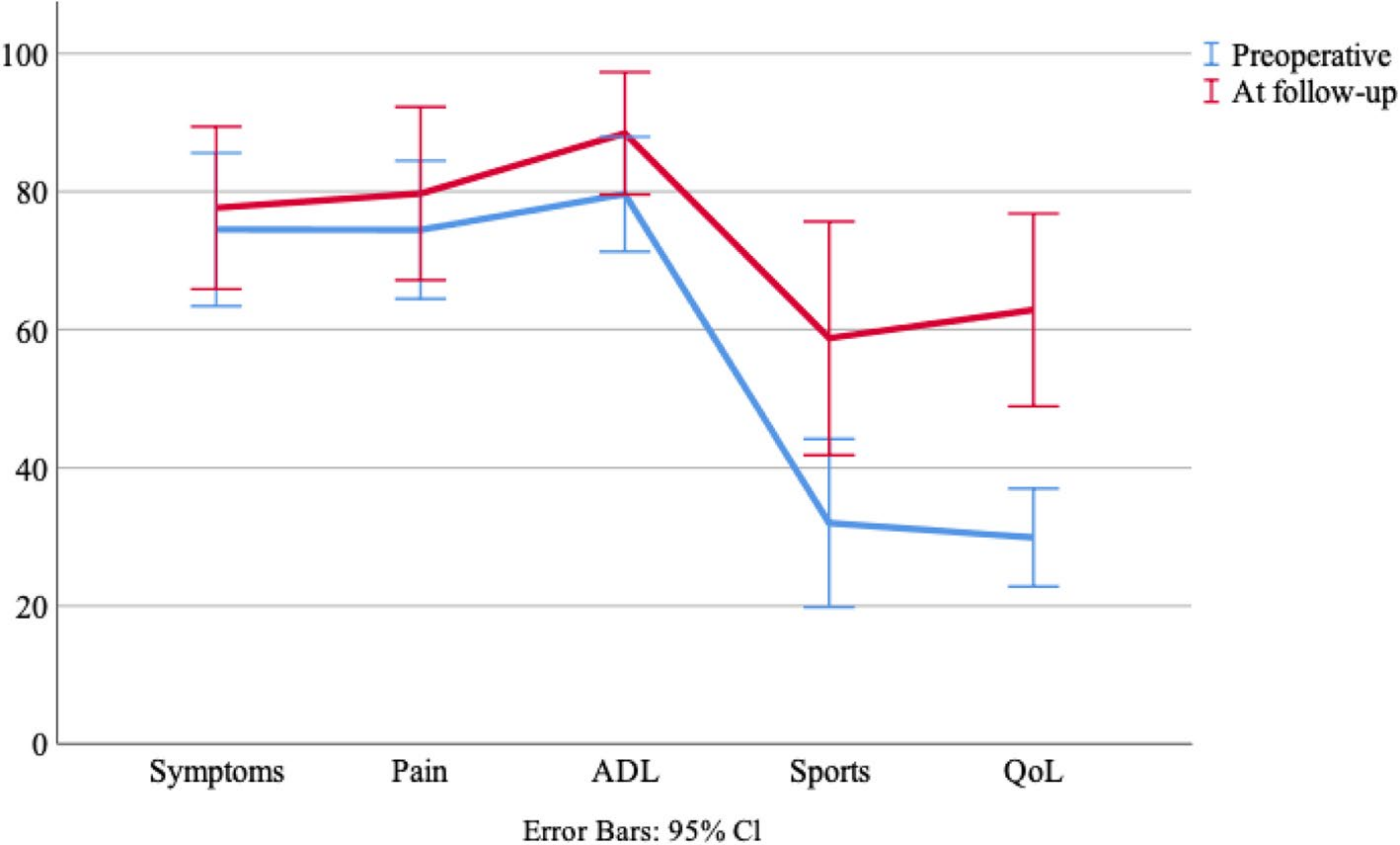
Preoperative and follow-up Knee injury and Osteoarthritis Outcome Score (KOOS) (mean (95% CI))

Figure 4 presents the preoperative KOOS and the KOOS at follow-up. The improvement was statistically significant regarding knee related QoL, *p* = 0.001.

Concerning general satisfaction, 19 out of 21 patients (90%) reported that they would have undergone surgery again had they known the outcome in advance. One patient was ambivalent, and one patient would not have undergone the surgery again knowing the outcome.

There were no deep postoperative nor other immediate postoperative complications after the allograft procedures. Four out of 21 patients had subsequent surgery in the same knee; three patients underwent screw removals due to discomfort and pain. The last patient has had multiple surgical procedures due to a pseudarthrosis of the patella not associated with the allograft procedure.

## Discussion

The main findings of this study include an improvement from the preoperative- to the follow-up score in the knee-related QoL KOOS subscale. Ninety percent of the patients said they would have had the surgery again had they known the outcome. There were no deep postoperative infections.

There was a wide variation in injury mechanism. This also reflects the diversity and differences between the patients included in this study. The majority sustained injuries during skiing and team sports such as soccer and handball, which are among the most popular sports practiced in our country [33].

All patients receiving allografts as primary reconstruction had a multiligament injury. These patients therefore required multiple grafts. The patients were usually given the choice between allografts or autografts from the uninjured contralateral knee. Allografts are often used for PCL reconstruction. PCL reconstruction can be performed as a double bundle reconstruction because it has two functional bundles, the anterolateral bundle and the smaller posteromedial bundle [31]. An Achilles allograft can be a good option for a double bundle reconstruction, however, in the present study, due to lack of convincing scientific evidence, only single bundle reconstructions were performed. Additionally, PCL rupture is often a part of a multiligament injury [37].

In multiligament injuries, acute surgery is normally performed within the first two weeks after injury [14]. All patients in this study had surgery performed after four weeks. The grafts cannot be shipped before the recipient’s name is known [40]. Storing the grafts at the hospital, with unknown recipient, is not possible due to the legislation in Belgium. This precludes the use of allografts for acute multiligament surgery as graft delivery can take up to four weeks. Delivery time varies depending on the graft type.

Current literature still discusses the timing of surgery, acute or chronic, to achieve the best possible outcome in multiligament patients. Levy et al. [22] did a systematic review including five studies which looked at the differences between acute and chronic surgery. In one of the studies, with patients having surgery in the acute phase had a higher average Lysholm functional score and higher activity level than those who had surgery after four weeks or more. Another systematic review with 24 studies found that chronic reconstruction could potentially produce the same results regarding stability compared to acute surgery [30]. The study also found that patients who underwent acute surgery presented with a limited ROM. Early mobilization seemed to improve the ROM for these patients [30]. The ROM examination in our study showed nearly normal values at the follow-up, and all patients underwent surgery in the chronic phase between five and forty months after injury. The majority had no or very little extension and flexion deficit. Other studies [4, 6, 19] also showed that the patients ROM recovered satisfactorily, although in all of the studies some patients were in need of arthroscopic adhesiolysis and/or manipulation under general anesthesia to achieve a satisfactory ROM.

Laxity, measured with the Lachman test, the pivot shift test, the posterior drawer test and the sag test, showed that most of the patients had relatively stable ligaments on examination at follow-up. Billières et al. [3] found that using non-irradiated allografts in reconstruction of all injured ligaments in multi-ligament knee injury is effective and safe. They also found that restoring satisfactory anteroposterior stability was difficult as five of eighteen patients (28%) had severely abnormal or abnormal laxity after ACL reconstruction. Nine of sixteen patients (56%) had abnormal or moderately to severely abnormal posterior laxity after PCL reconstruction. A study by Belk et al. [2] found that patients receiving autografts had decreased anteroposterior knee laxity postoperatively. However, improvement in clinical outcomes could be expected with both allograft and autograft in PCL reconstruction. Only two of the patients in our study had a higher grade of laxity on the Lachman test and the posterior drawer test. These patients seemed to be unaffected by this in their daily life and were satisfied with their results. Only one patient would not have undergone the procedure having known the outcome beforehand.

The Tegner score and knee related QoL KOOS at follow-up improved compared with pre-operative values. Visual evaluation of the KOOS figure shows a tendency of improvement for all five subscales. This implies that surgery was beneficial regarding the patient's level of activity. Being able to participate in physical activities may have a great influence on the quality of life. This statement is supported by the reported improved knee-related quality of life scores in the KOOS-scores before and after surgery. A study by Billières et al. [3] concluded that patients returned to their daily activities and sometimes to their sports activities at the same preinjury level after reconstruction of multiple ligaments. Multiple studies [9, 10, 19] found significant improvement in knee function and return to activity for many patients with chronic surgical management of the multiligament knee injury. Lind et al. [23] presented similar results as our study in improving the KOOS subscales at the follow-up. They compared preoperative KOOS and 1 year follow up KOOS of patients with multiligament injury, and found a significant improvement in KOOS, especially regarding sport function and QoL. Other studies [3, 41] which looked at clinical outcomes for patients receiving surgery for their multiligament knee injury found similar postoperative KOOS and Lysholm scores as presented in this study.

Many of the patients in this study had multiligament injuries with additional injuries like fractures and nerve injuries. These factors may affect the results of the PROMs negatively, making it difficult to differentiate what is related to the knee injury and what is related to other injuries. One patient had a patellar pseudarthrosis acquired prior to the surgery with allograft. This patient underwent three surgical procedures for the patellar issues after the allograft surgery and presented with low PROMs even though he had no obvious symptoms associated with the ligament reconstruction itself.

Multiligament injuries are often a consequence of high energy traumas which result in worse outcomes. Bearing in mind that most patients in this study had multiligament injuries, the clinical outcomes were satisfactory.

None of the patients reported immediate postoperative complications. Three patients, however, underwent screw removal after the primary procedure. In a study by Cook et al. [6] with 133 multiligament knee injuries in 130 patients, six patients underwent hardware and/or suture removal after the initial surgery. In another study [21], 23 knees in 22 patients of 458 ACL reconstructions experienced persistent pain and underwent screw removal. In all except two patients, autograft was used. In a study by Billières et al. [3], five out of twenty patients (25%) needed partial removal of hardware after reconstruction of multiligament knee injuries using allografts. It is relatively safe to assume that the need to remove screws is not limited to the use of allograft tissue. The slightly higher percentage of patients with this problem in our study may be due to a small number of patients participating in the study.

No reruptures of the allografts were reported at follow up. Much of today's literature reports a higher revision rate following allograft reconstruction compared to autografts [18, 25, 26, 44]. Using fresh frozen, non-radiated allografts generates the same results as for autografts, while radiated grafts seem to give higher failure rates than autografts [43]. A study by Mohan et al. [29] found a failure rate of 4.1% in patients who underwent autograft reconstruction, and 3.6% of those with allograft reconstruction, with no significant differences between the two. As results are discordant whether autograft or allograft provides better clinical outcomes in ACL reconstructions, Mascarenhas et al. [28] did a systematic review of meta-analyses that compared the two and found that there is evidence supporting no differences in rupture rates and clinical outcomes. On the other hand, a study by Tisherman et al. [39] found evidence supporting that allograft gave an increased risk of graft failure compared to autograft.

There were no deep infections in the present study. A retrospective study by Schuster et al. [35] analyzing 866 PCL and multiligament reconstructions, postoperative septic arthritis was found in four cases, only one of which had an allograft. A study by Katz et al. [20] compared the incidence of bacterial infection in ACL reconstructions with allograft versus autograft and did not find higher rate of deep bacterial infection when allograft tissue was used. Several other studies found no higher infection rate when using allograft tissue [1, 5, 12, 17]. Most of the current literature investigates infection in ACL reconstructions with allograft. There is little research on the incidence of postoperative infection when using allograft for multiligament injuries.

This study has several limitations, the most important of which is that it is a retrospective study without randomization, without any control group treated non-operatively and the limited number of patients. Some patients also underwent surgery with a combination of allografts and autografts. The patient group is very heterogeneous, both in terms of their demographics, and in terms of the type of injuries. Some patients had single ligament injury and others had multiligament injuries. Additional injuries vary between patients and the mechanism of injury, from high energy trauma to falling. Some patients had additional injuries such as fractures, injuries to the menisci, cartilage, and nerves. All these factors make the comparison of the clinical outcome challenging.

One strength of the study is that all patients underwent surgery at the same hospital and operations were performed by the same surgeon. The follow-up examination was performed by independent observers not involved in the surgical procedures.

## Conclusion

This study evaluated the outcomes after knee ligament reconstruction with allografts and found an improvement in knee-related quality of life. 90% of the patients would have had the surgery again and there were no deep postoperative infections.

## Data Availability

The datasets used and/or analyzed during the current study are available from the corresponding author on reasonable request.

## Abbreviations

ROM: Range of motion
PROM: Patient-reported outcome measures
KOOS: Knee injury and Osteoarthritis Outcome Score
ACL: Anterior Cruciate Ligament
LCL: Lateral collateral ligament
MCL: Medial collateral ligament
PCL: Posterior cruciate ligament
NKLR: The Norwegian National Knee Ligament Registry
BPTB: Bone-patellar tendon-bone
QoL: Quality of Life
REC: The Regional Committee for Medical and Health Research Ethics

## References

[1] Barker JU, Drakos MC, Maak TG, Warren RF, Williams RJ, Allen AA (2010). Effect of graft selection on the incidence of postoperative infection in anterior cruciate ligament reconstruction. Am J Sports Med.

[2] Belk JW, Kraeutler MJ, Purcell JM, McCarty EC (2018). Autograft versus allograft for posterior cruciate ligament reconstruction: an updated systematic review and meta-analysis. Am J Sports Med.

[3] Billieres J, Labruyere C, Steltzlen C, Gonzalez A, Boisrenoult P, Beaufils P, et al. (2019) Multiligament knee injuries treated by one-stage reconstruction using allograft: Postoperative laxity assessment using stress radiography and clinical outcomes. Orthop Traumatol Res 106(5):937-944 10.1016/j.otsr.2019.08.001 31494067

[4] Bin SI, Nam TS (2007). Surgical outcome of 2-stage management of multiple knee ligament injuries after knee dislocation. Arthroscopy.

[5] Condello V, Zdanowicz U, Di Matteo B, Spalding T, Gelber PE, Adravanti P (2019). Allograft tendons are a safe and effective option for revision ACL reconstruction: a clinical review. Knee Surg Sports TraumatolArthrosc.

[6] Cook S, Ridley TJ, McCarthy MA, Gao Y, Wolf BR, Amendola A (2015). Surgical treatment of multiligament knee injuries. Knee Surg Sports TraumatolArthrosc.

[7] Dedmond BT, Almekinders LC (2001). Operative versus nonoperative treatment of knee dislocations: a meta-analysis. The American journal of knee surgery.

[8] Dennis JA, Martinez OV, Landy DC, Malinin TI, Morris PR, Fox WP (2011). A comparison of two microbial detection methods used in aseptic processing of musculoskeletal allograft tissues. Cell Tissue Banking.

[9] Fanelli GC, Edson CJ (2004). Combined posterior cruciate ligament-posterolateral reconstructions with Achilles tendon allograft and biceps femoris tendon tenodesis: 2- to 10-year follow-up. Arthroscopy.

[10] Fanelli GC, Giannotti BF, Edson CJ (1996). Arthroscopically assisted combined posterior cruciate ligament/posterior lateral complex reconstruction. Arthroscopy.

[11] Gifstad T, Foss OA, Engebretsen L, Lind M, Forssblad M, Albrektsen G (2014). Lower risk of revision with patellar tendon autografts compared with hamstring autografts: a registry study based on 45,998 primary ACL reconstructions in Scandinavia. Am J Sports Med.

[12] Greenberg DD, Robertson M, Vallurupalli S, White RA, Allen WC (2010). Allograft compared with autograft infection rates in primary anterior cruciate ligament reconstruction. J Bone Joint Surg Am.

[13] Guo L, Yang L, Duan XJ, He R, Chen GX, Wang FY (2012). Anterior cruciate ligament reconstruction with bone-patellar tendon-bone graft: comparison of autograft, fresh-frozen allograft, and gamma-irradiated allograft. Arthroscopy.

[14] Harner CD, Waltrip RL, Bennett CH, Francis KA, Cole B, Irrgang JJ (2004). Surgical management of knee dislocations. J Bone Joint Surg Am.

[15] Howells NR, Brunton LR, Robinson J, Porteus AJ, Eldridge JD, Murray JR (2011). Acute knee dislocation: an evidence based approach to the management of the multiligament injured knee. Injury.

[16] Hulet C, Sonnery-Cottet B, Stevenson C, Samuelsson K, Laver L, Zdanowicz U (2019). The use of allograft tendons in primary ACL reconstruction. Knee Surg Sports TraumatolArthrosc.

[17] Indelli PF, Dillingham M, Fanton G, Schurman DJ (2002). Septic arthritis in postoperative anterior cruciate ligament reconstruction. ClinOrthopRelat Res.

[18] Kaeding CC, Pedroza AD, Reinke EK, Huston LJ, Spindler KP (2015). Risk factors and predictors of subsequent ACL injury in either knee after ACL reconstruction: prospective analysis of 2488 primary ACL reconstructions from the MOON cohort. Am J Sports Med.

[19] Karataglis D, Bisbinas I, Green MA, Learmonth DJ (2006). Functional outcome following reconstruction in chronic multiple ligament deficient knees. Knee Surg Sports TraumatolArthrosc.

[20] Katz LM, Battaglia TC, Patino P, Reichmann W, Hunter DJ, Richmond JC (2008). A retrospective comparison of the incidence of bacterial infection following anterior cruciate ligament reconstruction with autograft versus allograft. Arthroscopy.

[21] Kurzweil PR, Frogameni AD, Jackson DW (1995). Tibial interference screw removal following anterior cruciate ligament reconstruction. Arthroscopy.

[22] Levy BA, Dajani KA, Whelan DB, Stannard JP, Fanelli GC, Stuart MJ (2009). Decision making in the multiligament-injured knee: an evidence-based systematic review. Arthroscopy.

[23] Lind M, Nielsen TG, Behrndtz K (2018). Both isolated and multi-ligament posterior cruciate ligament reconstruction results in improved subjective outcome: results from the Danish Knee Ligament Reconstruction Registry. Knee Surg Sports TraumatolArthrosc.

[24] Lysholm J, Gillquist J (1982). Evaluation of knee ligament surgery results with special emphasis on use of a scoring scale. Am J Sports Med.

[25] Maletis GB, Chen J, Inacio MCS, Love RM, Funahashi TT (2017). Increased risk of revision after anterior cruciate ligament reconstruction with soft tissue allografts compared with autografts: graft processing and time make a difference. Am J Sports Med.

[26] Maletis GB, Inacio MC, Desmond JL, Funahashi TT (2013). Reconstruction of the anterior cruciate ligament: association of graft choice with increased risk of early revision. Bone Joint J.

[27] Malinin TI, Buck BE, Temple HT, Martinez OV, Fox WP (2003). Incidence of clostridial contamination in donors' musculoskeletal tissue. J Bone Joint Surg British.

[28] Mascarenhas R, Erickson BJ, Sayegh ET, Verma NN, Cole BJ, Bush-Joseph C (2015). Is there a higher failure rate of allografts compared with autografts in anterior cruciate ligament reconstruction: a systematic review of overlapping meta-analyses. Arthroscopy.

[29] Mohan R, Webster KE, Johnson NR, Stuart MJ, Hewett TE, Krych AJ (2018). Clinical outcomes in revision anterior cruciate ligament reconstruction: a meta-analysis. Arthroscopy.

[30] Mook WR, Miller MD, Diduch DR, Hertel J, Boachie-Adjei Y, Hart JM (2009). Multiple-ligament knee injuries: a systematic review of the timing of operative intervention and postoperative rehabilitation. J Bone Joint Surg Am.

[31] Pache S, Aman ZS, Kennedy M, Nakama GY, Moatshe G, Ziegler C (2018). Posterior cruciate ligament: current concepts review. Arch Bone JtSurg.

[32] Peskun CJ, Whelan DB (2011). Outcomes of operative and nonoperative treatment of multiligament knee injuries: an evidence-based review. Sports Med Arthrosc Rev.

[33] Nasjonal kompetanstjeneste for leddproteser og hoftebrudd (2017) 234-235. Rapport Nasjonalt Kårsbåndregister, Norway

[34] Roos EM, Roos HP, Lohmander LS, Ekdahl C, Beynnon BD (1998). Knee Injury and Osteoarthritis Outcome Score (KOOS)--development of a self-administered outcome measure. J Orthop Sports PhysTher.

[35] Schuster P, Gesslein M, Mayer P, Schlumberger M, Mayr R, Richter J (2018). Septic arthritis after arthroscopic posterior cruciate ligament and multi-ligament reconstructions is rare and can be successfully treated with arthroscopic irrigation and debridement: analysis of 866 reconstructions. Knee Surg Sports TraumatolArthrosc.

[36] Singh R, Singh D, Singh A (2016). Radiation sterilization of tissue allografts: A review. World J Radiol.

[37] Strauss MJ, Varatojo R, Boutefnouchet T, Condello V, Samuelsson K, Gelber PE (2019). The use of allograft tissue in posterior cruciate, collateral and multi-ligament knee reconstruction. Knee Surg Sports TraumatolArthrosc.

[38] Tegner Y, Lysholm J (1985). Rating systems in the evaluation of knee ligament injuries. ClinOrthopRelat Res.

[39] Tisherman R, Wilson K, Horvath A, Byrne K, De Groot J, Musahl V (2019). Allograft for knee ligament surgery: an American perspective. Knee Surg Sports TraumatolArthrosc.

[40] Union E. Commission directive 2006/86/EC. journal. 2006 [cited 2019 25.11.2019]; 49(L294):[1–80 pp.]. Available from: https://eur-lex.europa.eu/LexUriServ/LexUriServ.do?uri=OJ:L:2006:294:0032:0050:EN:PDF.

[41] Wajsfisz A, Bajard X, Plaweski S, Djian P, Demey G, Limozin R (2014). Surgical management of combined anterior or posterior cruciate ligament and posterolateral corner tears: for what functional results?. OrthopTraumatolSurg Res.

[42] Wang HD, Zhang H, Wang TR, Zhang WF, Wang FS, Zhang YZ (2018). Comparison of clinical outcomes after anterior cruciate ligament reconstruction with hamstring tendon autograft versus soft-tissue allograft: A meta-analysis of randomised controlled trials. Int J Surg (London, England).

[43] Wang S, Zhang C, Cai Y, Lin X (2018). Autograft or Allograft? Irradiated or Not? A contrast between autograft and allograft in anterior cruciate ligament reconstruction: a meta-analysis. Arthroscopy.

[44] Wasserstein D, Khoshbin A, Dwyer T, Chahal J, Gandhi R, Mahomed N (2013). Risk factors for recurrent anterior cruciate ligament reconstruction: a population study in Ontario, Canada, with 5-year follow-up. Am J Sports Med.

